# Wide‐scale comparative analysis of longevity genes and interventions

**DOI:** 10.1111/acel.12659

**Published:** 2017-08-24

**Authors:** Hagai Yanai, Arie Budovsky, Thomer Barzilay, Robi Tacutu, Vadim E. Fraifeld

**Affiliations:** ^1^ The Shraga Segal Department of Microbiology, Immunology and Genetics Center for Multidisciplinary Research on Aging Ben‐Gurion University of the Negev POB 653 Beer Sheva 8410501 Israel; ^2^ Biotechnology Unit Technological Center Beer Sheva 8489101 Israel; ^3^ Computational Biology of Aging Group Institute of Biochemistry Romanian Academy Bucharest 060031 Romania; ^4^ Chronos Biosystems SRL Bucharest Romania

**Keywords:** comparative analysis, evolutionary conservation, gene enrichment, gene orthology, longevity genes, proteome, public and private mechanisms of aging/longevity

## Abstract

Hundreds of genes, when manipulated, affect the lifespan of model organisms (yeast, worm, fruit fly, and mouse) and thus can be defined as longevity‐associated genes (LAGs). A major challenge is to determine whether these LAGs are model‐specific or may play a universal role as longevity regulators across diverse taxa. A wide‐scale comparative analysis of the 1805 known LAGs across 205 species revealed that (i) LAG orthologs are substantially overrepresented, from bacteria to mammals, compared to the entire genomes or interactomes, and this was especially noted for essential LAGs; (ii) the effects on lifespan, when manipulating orthologous LAGs in different model organisms, were mostly concordant, despite a high evolutionary distance between them; (iii) LAGs that have orthologs across a high number of phyla were enriched in translational processes, energy metabolism, and DNA repair genes; (iv) LAGs that have no orthologs out of the taxa in which they were discovered were enriched in autophagy (Ascomycota/Fungi), G proteins (Nematodes), and neuroactive ligand–receptor interactions (Chordata). The results also suggest that antagonistic pleiotropy might be a conserved principle of aging and highlight the importance of overexpression studies in the search for longevity regulators.

## Introduction

The role of genetic factors in determination of longevity and aging patterns is an intensively studied issue (Vijg & Suh, 2005; Kenyon, 2010). Hundreds of genes, when manipulated, have been shown to affect the lifespan of model organisms (yeast, worm, fruit fly, and mouse) (Tacutu *et al*., 2013). These genes (further denoted as longevity‐associated genes, LAGs) could be defined as those whose modulation of function or expression (such as gene knockout, overexpression, partial or full loss‐of‐function mutations, RNA interference, and genetic polymorphisms) results in noticeable changes in longevity—lifespan extension or accelerated aging (Budovsky *et al*., 2007; Tacutu *et al*., 2013).

We have previously investigated the characteristic features of LAGs and found that (i) they display a marked diversity in their basic function and primary cellular location of the encoded proteins (Budovsky *et al*., 2007); and (ii) LAG‐encoded proteins display a high connectivity and interconnectivity. As a result, they form a scale‐free protein–protein interaction network (‘longevity network’), indicating that LAGs could act in a cooperative manner (Budovsky *et al*., 2007; Wolfson *et al*., 2009; Tacutu *et al*., 2010a, 2011, 2012). (iii) Many LAGs, particularly those that are hubs in the ‘longevity network’, are involved in age‐related diseases (including atherosclerosis, type 2 diabetes, cancer, and Alzheimer's disease), and in aging‐associated conditions (such as oxidative stress, chronic inflammation, and cellular senescence) (Budovsky *et al*., 2007, 2009; Wolfson *et al*., 2009; Tacutu *et al*., 2010a, 2011). (iv) The majority of LAGs established by that time in yeast, worms, flies, and mice have human orthologs, indicating their conservation ‘from yeast to humans’ (Budovsky *et al*., 2007, 2009). This assumption was also supported by studies on specific LAGs or pathways such as Foxo (Martins *et al*., 2016), insulin/IGF1/mTOR signaling (Tatar *et al*., 2003; Warner, 2005; Piper *et al*., 2008; Ziv & Hu, 2011; Gems & Partridge, 2013; Zhang & Liu, 2014; Pitt & Kaeberlein, 2015), Gadd45 (Moskalev *et al*., 2012), and cell–cell and cell–extracellular matrix interaction proteins (Wolfson *et al*., 2009). Again, the above studies were limited only to the four model organisms and humans.

Now, the existing databases on orthologs allow for an essential extension of the analysis of LAG orthology, far beyond the traditional model organisms and humans. In particular, the data deposited in the InParanoid database—Eukaryotic Ortholog Groups (http://inparanoid.sbc.su.se/, Sonnhammer & Ostlund, 2015) include orthologs for the complete proteomes of 273 species. Here, we report the results of an unprecedentedly wide‐scale analysis of 1805 LAGs established in model organisms (available at Human Ageing Genomic Resources (HAGR)—GenAge database; http://genomics.senescence.info/genes/longevity.html, Tacutu *et al*., 2013), with regard to their putative relevance to public and private mechanisms of aging.

## Results

### Orthology of longevity‐associated genes

Our first question was how LAGs orthologs are distributed across diverse taxonomic groups. For that purpose, we extracted the LAG orthologs for all the species in the InParanoid database, using a software developed in our laboratory (see Methods). For each gene of interest, the evolutionary conservation was evaluated as the presence or absence of orthologs across 205 proteomes (all species available excluding parasites) for a high InParanoid score of 1.0. Parasites were excluded from the analysis because they usually keep the minimal set of genes required for survival in the hosts, and thus, their inclusion could bias the results into overstating the conservation of these genes and diminish the conservation of others.

**Figure 1:**
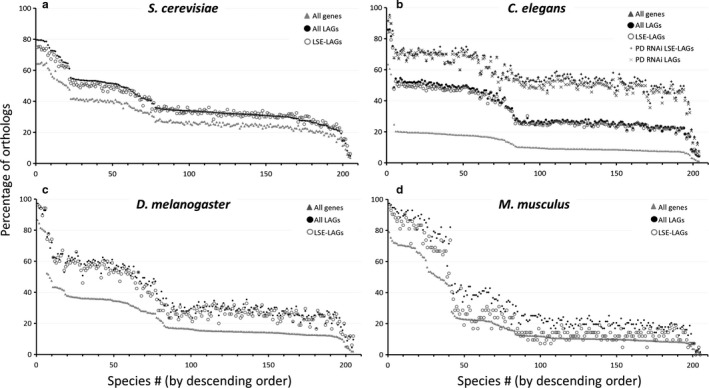
Percentage of orthologs of longevity‐associated genes (LAGs) from the four model organisms across 205 species. Each graph represents one of the four model organisms and the LAGs discovered for that species. Each dot represents the percentage of orthologs between the model species and a single other species (total of 205 species from all Kingdoms; for a full list of species see Table S1). The entire proteome of the model species (extracted from the InParanoid database) was used as control. The species (X‐axis) are ordered in descending order of the percentage of orthologs for the entire proteome. Presented are the ortholog percentage of the entire proteome (gray triangle), LAGs (black circle), LAGs discovered by lifespan extension (LSE‐LAGs, gray circle), *Caenorhabditis elegans* essential LAGs discovered by postdevelopmental RNAi (PD RNAi LAGs, gray x), and *C. elegans* essential LAGs discovered by postdevelopmental RNAi that resulted in lifespan extension (PD RNAi LSE‐LAGs, black +). (a) *Saccharomyces cerevisiae, n *= 6590 for control, 824 for all LAGs, and 277 for LSE‐LAGs. (b) *C. elegans, n *= 20 325 for control, 733 for all LAGs, 491 for LSE‐LAGs, 127 for PD RNAi LAGs, and 107 for PD RNAi LSE‐LAGs. (c) *Drosophila melanogaster*,* n *= 13 250 for control, 136 for all LAGs, and 85 for LSE‐LAGs. (d) *Mus musculus, n *= 21 895 for control, 112 for all LAGs, and 42 for LSE‐LAGs. The vast majority of pairwise differences between LAGs and the entire proteome are significant (*P* < 0.05), with a few exceptions of fringe cases as described in the text. For most *M*. *musculus* LSE‐LAGs, the pairwise differences are insignificant (*P* > 0.05), with a few exceptions where the number of orthologs was relatively high.

As seen in Fig. 1, for the vast majority of InParanoid species, the fraction of conserved genes was significantly higher for LAGs than for the entire proteome of the same model organism. The few exceptions were fringe cases where the baseline orthology was either very high (phylogenetically very close species, for example, *Caenorhabditis elegans* and *Caenorhabditis briggsae*), or very low (phylogenetically very distant species, for example, *Mus musculus* and *Korarchaeum cryptofilum*) (Table S1).

**Figure 2:**
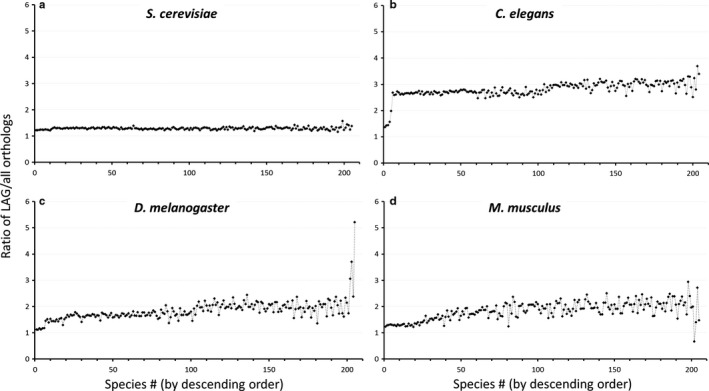
Ratio of LAGs orthologs to the entire proteome. Each graph represents the LAGs discovered in the indicated model species. Each dot represents the ratio between the number of LAG orthologs to the orthologs from the entire proteome, for a single other species (total of 205 species from all Kingdoms; for a full list of species see Table S1). The species (X‐axis) are ordered in descending order of ortholog percentage for the entire proteome. (a) *Saccharomyces cerevisiae, n *= 6590 for control and 824 for LAGs; (b) *Caenorhabditis elegans, n *= 20 325 for control and 733 for LAGs; (c) *Drosophila melanogaster*,* n *= 13 250 for control and 136 for LAGs; (d) *Mus musculus, n *= 21 895 for control and 112 for LAGs.

Remarkably, despite the high diversity of the species under analysis, the ratio between the LAG orthologs and the orthologs of the entire proteome was relatively constant along the evolutionary axis (Fig. 2). This could indicate that the high conservation of LAGs is relatively independent of evolutionary distance.

As lifespan extension experiments can be regarded as more robust, we placed a special focus on LAGs that extended lifespan, when manipulated (LSE‐LAGs). The results indicate that the major principles still hold (Fig. 1). For the three lower model organisms (*S. cerevisiae, C. elegans, D. melanogaster*), the distribution of the LSE‐LAGs orthologs was almost identical to all LAGs; a clear trend was also observed for *M. musculus*, although it did not reach a statistically significant value, most likely because of the relatively low number of lifespan‐extending interventions in this model.

It should be taken into account that genes which *a priori* have orthologs in humans and are involved in basic biological processes or major diseases are more often tested for their potential effect on lifespan. Despite this obvious bias, an important point is that among the model organisms examined, the highest conservation ratio was observed for *C. elegans* (*P *< E‐8 for all comparisons; Fig. 2a), where the majority of LAGs were identified by means of an unbiased genome‐wide RNA interference (RNAi) screens (Lee *et al*., 2003; Hamilton *et al*., 2005; Hansen *et al*., 2005; Yanos *et al*., 2012).

Postdevelopmental gene inactivation using RNAi is of special interest. This is because it allows for discovering longevity regulators that could not be discovered otherwise, because their predevelopmental inactivation causes a lethal phenotype (Tacutu *et al*., 2012). According to WormBase (http://www.wormbase.org/; Howe *et al*., 2016), 127 of the 733 known *C. elegans* LAGs are essential for development and growth, which means that worm LAGs are enriched in essential genes by approximately fivefold compared to the entire genome (Tacutu *et al*., 2012). This is even more pronounced among LAGs that extend worm lifespan by more than 20% when inactivated: They are enriched 15‐fold in essential genes. As essential genes are generally more evolutionary conserved than nonessential ones (Tacutu *et al*., 2011), we looked at the ortholog distribution of the 127 essential worm LAGs and found that they are indeed dramatically more conserved than all LAGs (Fig. 1b). The same is also true for essential LAGs where the postdevelopmental RNAi resulted in lifespan extension (Fig. 1b). Remarkably, postdevelopmental inactivation of worm essential LAGs has been shown to predominantly extend lifespan rather than reduce it (Curran & Ruvkun, 2007; Tacutu *et al*., 2012), which means that they have detrimental effects later in life. As these genes are essential for the early stages of life, but their postdevelopmental inactivation resulted in lifespan extension, this by definition is consistent with Williams's idea of antagonistic pleiotropy (Williams 1957). In support of this notion are also our previous studies (Budovsky *et al*., 2007, 2009; Tacutu *et al*., 2010b, 2011, 2012) and the study of Promislow (2004). All in all, the results suggest that antagonistic pleiotropy might be a conserved principle of aging.

One of the strong features of InParanoid is that it provides the best balance between sensitivity and specificity (Chen *et al*., 2007). Yet, the proteomes found in the InParanoid database contain many poorly annotated proteins and predicted transcripts that were not experimentally verified (Sonnhammer & Ostlund, 2015). These proteins have relatively few orthologs in other species and therefore could influence the results. In contrast, the interactomes from the BioGrid database (http://www.thebiogrid.org), the largest repository of validated PPIs, almost exclusively include experimentally verified proteins (Chatr‐Aryamontri *et al*., 2015). Therefore, the BioGrid data could serve as an additional, high quality control for a more rigorous testing of the evolutionary conservation of LAGs. For this purpose, we used the interactomes of *S. cerevisae*,* C. elegans*, and *D. melanogaster*. As seen in Fig. S1, the same trend of over‐conservation of LAGs was also observed in comparison with the BioGrid control. Mouse was not included in the analysis because its BioGrid gene list still contains a relatively small portion of the entire genome and thus could not provide a reliable control.

Altogether, the results clearly show a high evolutionary conservation of LAGs across distant species. With regard to this, a question arises as to whether this observation is attributed to an enrichment of specific categories that are known to be strongly preserved in the course of evolution. From the available data on gene and protein annotations for the four model species, we noted that LAGs are enriched in genes that belong to categories known to be extraordinarily conserved in evolution, such as the ribosomal or mitochondrial genes (Table S2). However, exclusion of LAGs belonging to these categories from the analysis had almost no impact (Fig. S2). Therefore, we conclude that the high evolutionary conservation of LAGs is not solely attributed to an enrichment of proteins from exceptionally conserved categories, but rather reflects a general trend.

### ‘Public’ and ‘private’ LAG categories

**Figure 3:**
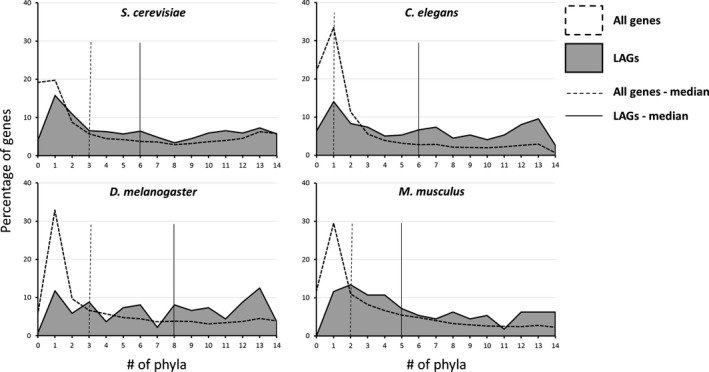
Distribution of LAGs according to the number of phyla in which LAGs have orthologs. Each graph represents the distribution of LAGs (gray area) discovered in the indicated model species. The entire proteome was used as a control (dotted line). X‐axis depicts the number of phyla in which the genes have orthologs. The medians of the distributions are presented as vertical lines: dotted line for all genes and smooth black line for LAGs.

The distinction between public and private mechanisms of aging and longevity is a fundamental question in comparative studies of biogerontology (Gems & Partridge, 2013). We attempted to shed some light on this subject based on the ortholog distribution of LAGs in different taxa. Yet, it is important to note that if a given LAG is highly evolutionary conserved, it does not automatically translate to its role in a public mechanism of aging. In fact, in order to draw conclusions on public or private mechanisms from the presence or lack of orthologs, one must (i) have a context on the mode of operation of a given protein as its function could differ between species; or (ii) compare groups of proteins belonging to a certain pathway or category, so that generalized assumptions may be made. The data used in this study only allow for the second approach. Thus, we comprised lists of proteins under different conservation criteria, for example, proteins that have orthologs across at least 12 phyla or have orthologs in a limited number of taxa (for more details, see Table S3). As shown in Fig. 3, LAG orthologs are distributed over more phyla than the entire genome, again indicating their wider evolutionary conservation. Nevertheless, while most LAGs are broadly presented across phyla, a considerable portion of them (around 10–20%) are specific to a relatively small number of phyla (Fig. 3).

To get further insight into the universality of longevity‐associated pathways, we carried out an enrichment analysis for LAGs with orthologs across a large number of phyla (‘public’) and those that are limited to specific phyla (‘private’). For the ‘private’ analysis, we used the phylum of the corresponding model organism (as depicted in the Table 1), as smaller taxonomic groups did not yield statistically conclusive results. The detailed results of the enrichment analysis are available in Table S4 and Fig. S3.

**Table 1:** ‘Public’ and ‘private’ enriched categories. The table depicts the most enriched categories for lists of proteins of all longevity‐associated genes (all LAGs) and LAGs discovered by either lifespan extension (LSE‐LAGs) or lifespan reduction (LSD‐LAGs), under different evolutionary conservation criteria (defined as the presence of orthologs across a listed number of phyla). For a detailed enrichment analysis, see Tables [Link], [Link], [Link]

Public/Private		*Saccharomyces cerevisiae*	*Caenorhabditis elegans*	*Drosophila melanogaster*	*Mus musculus*
Public	Taxa groups where orthologs are present	*at least 12 phyla*	*at least 12 phyla*	*at least 10 phyla*	*at least 10 phyla*
	All LAGs	Ribosome and translation Mitochondria Citrate cycle (TCA cycle)	Ribosome and translation Mitochondria Oxidative phosphorylation NADH activity	FoxO signaling Autophagy	DNA repair, especially Nucleotide excision repair
	LSE‐LAGs	Ribosome and translation	Ribosome and translation Mitochondria Oxidative phosphorylation	Development Oxidoreductase	No enrichment
	LSD‐LAGs	Ribosome and translation Mitochondria Citrate cycle (TCA cycle) Autophagy DNA repair	Autophagy	Autophagy FoxO signaling	DNA repair, especially Nucleotide excision repair
Private (indicated taxa)	Taxa groups where orthologs are present	*only in Fungi/Ascomycota*	*only in Nematoda*	*only in Arthropoda*	*only in Chordata*
	All LAGs	Autophagy	G protein related	No enrichment	Neuroactive ligand–receptor interaction
	LSE‐LAGs	Meiosis	G protein related	No enrichment	Neuroactive ligand–receptor interaction
	LSD‐LAGs	Autophagy Mitochondrion Mitophagy	Transcription regulation	No enrichment	No enrichment

Overall, the analysis of the most conserved (public) LAGs revealed that they, not surprisingly, fall under three major categories (Table 1): (i) ribosome and translational processes, (ii) mitochondria and energy metabolism pathways (including the FoxO pathway), and (iii) DNA repair. At a first view, it may seem that the most conserved LAGs are enriched for these categories just because of their function, regardless of their role in aging, that is, simply because they belong to very basic and therefore highly conserved biological processes. However, comparing the most conserved LAGs against different backgrounds showed that it is not the case. Indeed, these pathways were not only overrepresented when compared to the entire genome or to all LAGs, but—what is most important—when compared to all highly conserved genes (i.e., all genes with orthologs across at least 10 or 12 phyla; Table S4). The results provide a strong support for previous studies (in particular those by McElwee *et al*., 2007; Smith *et al*., 2008; Freitas *et al*., 2011; MacRae *et al*., 2015; Ma *et al*., 2016;) and highlight the public role of the above categories in the control of lifespan. It should however be noted that the number of LAGs was not always sufficient for a robust enrichment analysis, especially for the mouse and fly models (see Table S3); the results from the yeast and worm models were more significant and thus more reliable.

LAGs were discovered in either lifespan‐extending or lifespan‐reducing experiments (or sometimes both). Do these two groups display any specificity in their enriched pathways/categories? Not surprisingly, the common public categories for both groups included the ribosomal and mitochondrial genes (Table 1, Table S5 and S6). However, we surprisingly found that the division of all LAGs in such a way also revealed a distinct pattern of enrichment. In particular, an overrepresentation of autophagy‐related and DNA repair genes was observed only among the LAGs discovered in lifespan‐decreasing experiments (LSD‐LAGs; Table 1 and Table S5). In contrast, LAGs discovered by lifespan‐extending experiments (LSE‐LAGs; Table 1 and Table S6) were specifically enriched for oxidative phosphorylation and oxidoreductase.

Due to the high evolutionary conservation of LAGs, those that have orthologs only in the same phylum as the model species in which they were discovered are relatively small in number. Because of that, the enrichment analysis of these genes yielded less significant results (Table S4). Nevertheless, the ‘private’ list for *S. cerevisae* (i.e., yeast LAGs with orthologs only in Ascomycota/Fungi) was found to be enriched with autophagy‐related genes, which can be attributed mostly to LSD‐LAGs (Table 1). For LAGs that have orthologs only in Nematoda, we found enrichment in G protein‐related genes, apparently attributed to LSE‐LAGs. This is surprising because both autophagy and G protein signaling represent basic and highly conserved processes which were shown to be involved in aging and longevity in various model organisms (Lans & Jansen, 2007; Hahm *et al*., 2009; Rubinsztein *et al*., 2011; Schneider & Cuervo, 2014). Yet, the unusual enrichment of these pathways in yeast and worms definitely highlights their importance in determination of longevity for these taxa specifically, although it does not exclude their role in mechanisms of aging for higher taxa. For vertebrates, we found a significant enrichment of LSE‐LAGs in Neuroactive ligand–receptor interaction, which could reflect the importance of neuroendocrine regulation of aging and longevity in higher organisms (Dilman *et al*., 1986; Frolkis, 1988; Blagosklonny, 2013). All of the above results obtained by David were also validated by the WebGestalt, EnrichR and Panther tools (see Methods; *C*. *elegans* data are presented as an example in Fig. S3).

### Stress response genes and the importance of overexpression interventions

**Figure 4:**
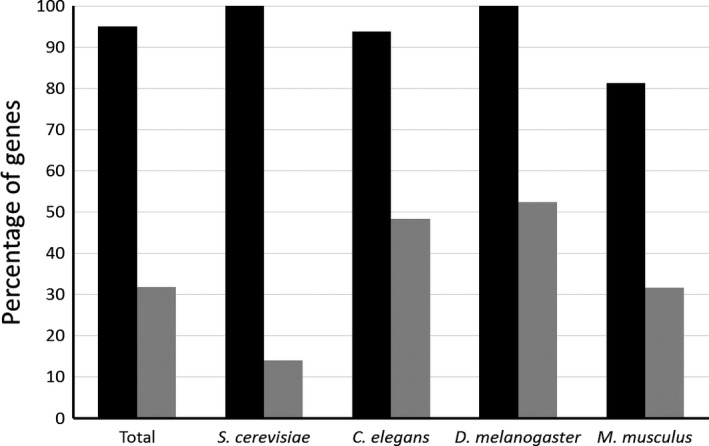
Percentage of manipulations on stress response LAGs that extended the lifespan. Only LAGs which were termed as ‘stress response’ under the GO classification system are included. Depicted is the percentage of overexpression (black) and knockout/knockdown (gray) interventions that resulted in lifespan extension. All intraspecies differences between the effects of overexpression and knockout/knockdown on lifespan were significant (*P* < 0.001). The full list of stress response LAGs is available in Table S7.

The vast majority of LAGs were discovered by downregulating gene activity (e.g., knockout and RNAi). For example, in *C. elegans*, only 52 of hundreds of LAGs were discovered by overexpression assays. This could potentially create a bias toward overestimation of certain categories for longevity regulation on the expanse of others. A good illustration of that point is that the ‘stress response’ category was noticeably absent from the enrichment analysis. Yet, it could be expected that many stress response genes would extend lifespan when upregulated rather than downregulated (Moskalev *et al*., 2014). Indeed, our analysis shows that overexpression of LAGs listed in the GO database as stress response genes (*n *= 81; Table S7) almost exclusively (95%) resulted in lifespan extension (Fig. 4). Of these LAGs, 19 were also tested by knockout or knockdown experiments. Remarkably, in 18 cases, this resulted in lifespan reduction and only in the case of Sirtuin‐1 in yeast—in lifespan extension. Apart from the longevity value of stress response genes, these observations clearly demonstrate the importance of overexpression experiments in longevity studies.

### Concordancy and discordancy in lifespan‐modulating genetic interventions

**Figure 5:**
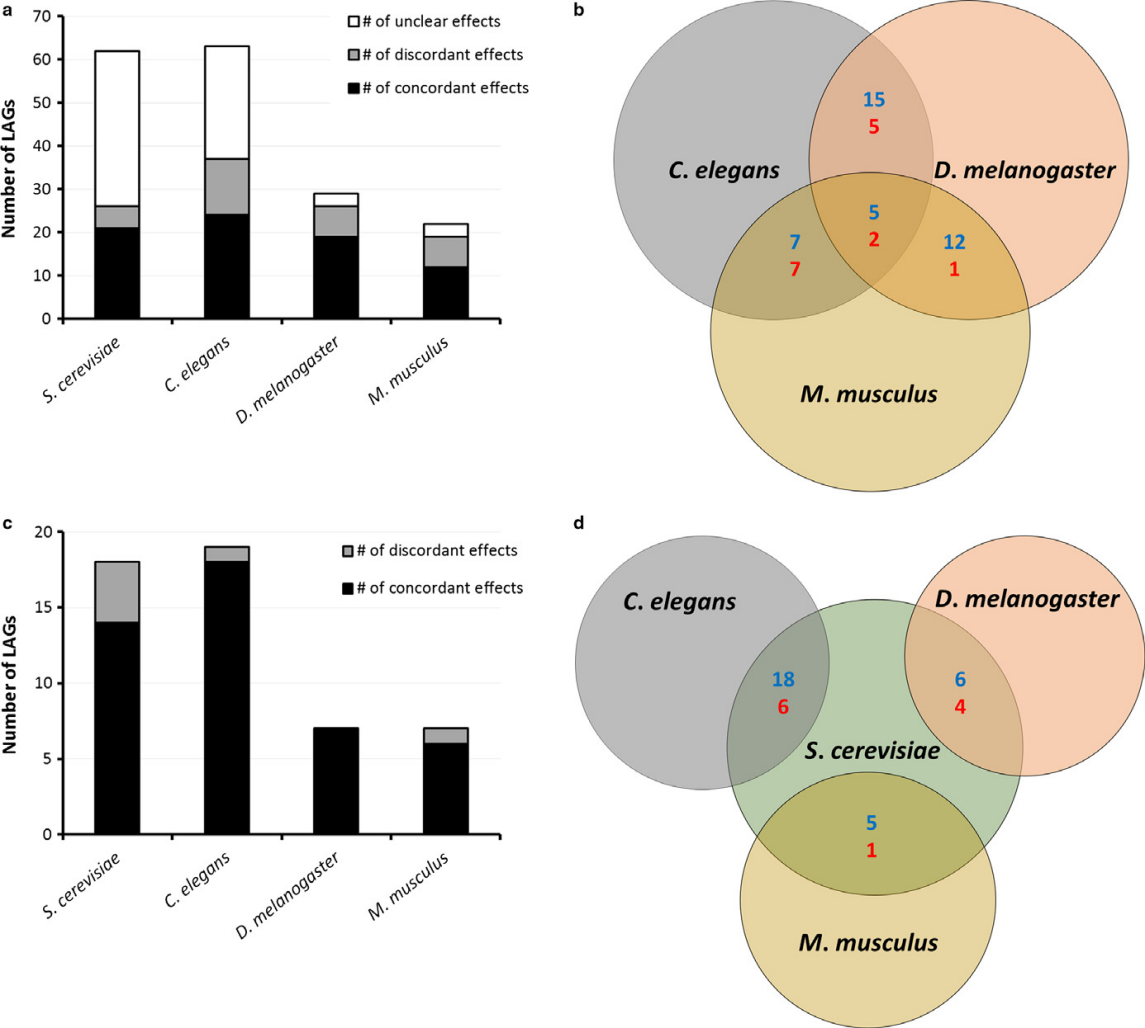
Concordancy in LAG manipulations across model organisms. Concordancy was determined according to the classification of LAGs as pro‐ or anti‐longevity genes (Tacutu *et al*., 2013). That is, if a given LAG was determined as a pro‐ or anti‐longevity gene in two or more species, it was termed ‘concordant’; otherwise, it was termed ‘discordant’. A detailed table is available in Table S8. (a) Summary of the concordancy for LAGs from each model species which have also been tested in two or more species (interspecies). (b) Venn diagram of the concordancy between species. (c+d) Summary of the concordancy of LAG manipulations within the same species (intraspecies).

Considering the conservation of many LAGs over a broad evolutionary distance, a valid question is whether modulating a given LAG in different species has a similar impact on longevity, that is, lifespan extension or reduction. This was previously addressed for worm and yeast, where the genetic component of lifespan determination was found to be significantly conserved (Smith *et al*., 2008). Here, we broadened the question to *all* available model organisms. Namely, we compared all orthologs which were shown to have an impact on longevity in more than one species. Overall, we found that approximately 10% of LAGs' orthologs (*n *= 184) were identified as such in at least two model organisms; 36 LAGs' orthologs were identified in three and 20 in four model organisms. The number of concordant effects was significantly higher than the discordant ones (*P* < 0.003). That is, manipulation of LAGs has, more than often, the same effect in different species (Fig. 5, Table S8). Unfortunately, a substantial portion of the genetic interventions in yeast and worms could not be clearly defined as concordant or discordant with other model organisms (Fig. 5a, white), mostly due to a major difference in methods and evaluation criteria (Tacutu *et al*., 2013).

When looking at pairwise comparisons (Fig. 5b), it is evident that the level of concordancy is very high for some pairs of species (for example, *M. musculus* and *D. melanogaster*) and lower for others (for example, *M. musculus* and *C. elegans*). In order to discern what could bring about this difference, we calculated a conservation index for each pair of orthologs (as previously described by Huang *et al*., 2004) and compared the results to the concordancy/discordancy of the effects. As seen in Fig. S4, the observed discordancy could not be explained by sequence dissimilarity. One of the possible explanations for the observed discordancy is that in these cases orthologous LAGs were discovered by interventions which greatly differ from one another (e.g., knockout and overexpression). As such, if a given LAG is knocked out and as a result the animal ages more rapidly, that LAG is defined as a ‘pro‐longevity’ gene; however, an overexpression of the same LAG will not necessarily increase lifespan. For example, a knockout of G protein, alpha subunit (*gpa‐9*) in *C. elegans* increases maximum lifespan by up to 50%, but paradoxically, its overexpression also increases the worm maximum lifespan (by 20%) (Schneider & Cuervo, 2014). If such a difference can occur in the same species, more so could be expected when testing for effects on lifespan between different model organisms. Indeed, as is evident from Fig. S5, the concordancy increased significantly (from 73% to 88%) when a *similar* intervention was performed. Then, at least some of the discordancy could be explained by a variety in the methods of intervention. It should however be noted that the vast majority of intraspecies comparisons of opposite interventions have brought about concordant effects (Fig. 5c), so that we cannot rule out interspecies differences that caused some of the inconsistencies.

Interestingly, only five LAGs (*Sod2*,* Sirt1*,* Mtor*,* Fxn*, and *Rps6 kb1*; in total, 20 orthologs) were tested for their impact on longevity in all four model species. The manipulations of these genes showed a predominantly concordant effect on longevity, with the exception of *Fxn* (*Frh‐1*) which has an opposite effect only in *C. elegans* (Table S7). Altogether, the results indicate a clear trend of concordancy in the effects of LAG manipulations across model species despite a high evolutionary distance between them. The much smaller portion of discordant cases could be attributed to either technical or biological issues, or both.

## Discussion

Our wide‐scale analysis of longevity‐associated genes (LAGs) shows that their orthologs are consistently overrepresented across diverse taxa, compared with the orthologs of other genes, and this conservation was relatively independent of evolutionary distance (Figs 1, 2, 3). The high evolutionary conservation was evident for LAGs discovered in all of the four major model organisms (yeast, *S. cerevisae*; worm, *C. elegans*; fly, *D. melanogaster*; mouse, *M. musculus*), but was especially relevant for *C. elegans,* where a large portion of LAGs were identified by genome‐wide screens, thus minimizing potential biases. Moreover, many worm LAGs were discovered by postdevelopmental RNAi on genes essential for growth and development, and this predominantly resulted in lifespan extension (Curran & Ruvkun, 2007; Tacutu *et al*., 2012). That is, postdevelopmental suppression of genes that are vital early in life but are detrimental later in life, can be beneficial for longevity. The orthologs of these LAGs are also highly overrepresented across diverse taxa. Altogether, the *C*. *elegans* analysis suggests that antagonistic pleiotropy might be a highly conserved principle of aging.

As one of Niven's laws states: ‘It is easier to destroy than to create’. Indeed, there are many more ways to make an organism live shorter than to make it live longer. The enrichment analysis demonstrated the difference in public and private pathways/categories, with potential importance for lifespan extension or development of an early aging phenotype (Table 1). It is worthwhile to note that while the enrichment analysis definitely highlights the importance of the overrepresented categories, it does not exclude the importance of nonenriched ones. For example, a well‐recognized longevity pathway, insulin/IGF signaling, does not appear as an enriched pathway in our analysis but has been previously shown to be a public mechanism of aging (Piper *et al*., 2008). Another important category that did not fall into the enriched ones is stress response genes. Stress resistance has long been linked to longevity in many animal models (Johnson *et al*., 2002; Moskalev *et al*., 2014). In this study, we have specifically addressed stress response genes and showed that their overexpression mostly results in lifespan extension (Fig. 4). This further emphasizes the importance of overexpression interventions in longevity studies, which should be a point for future investigations. The latter would not only drive the discovery of new longevity regulators but could also strengthen the validity of LAGs that were discovered by knockout or knockdown experiments, as we have shown in this study (Fig. 5c). The recent development of novel CRISPR‐based gene activation technologies could provide a strong platform and push toward this approach.

An important observation in our study was that the majority of manipulations on LAG orthologs in more than one model animal resulted in concordant effects on longevity (Fig. 5). This strengthens the paradigm of ‘public’ longevity pathways and of using model animals to study longevity, even across a large evolutionary distance. This notion is further strengthened when combined with the observation of Smith *et al*. (2008) who demonstrated that the existence of an ortholog is probably accompanied by a preserved role in longevity. Yet, we also observed LAGs with ortholog presence only in a limited number of taxa, or that displayed discordant effects when tested in more than one species (Fig. 5), which could, at least in part, be attributed to ‘private’ mechanisms of aging. Definitely, more comparative studies are warranted to better discriminate between private and public mechanisms, with unified methods of intervention and evaluation in mind. A recent study by Harel *et al*. (2015) could serve as a step in that direction by offering a new model of short‐lived vertebrate species. In perspective, the combination of the existing data on LAGs with the emerging data on their expression throughout lifespan could bring about a deeper understanding of the role of genetic factors in aging and longevity.

## Experimental procedures

### Gene lists

#### Longevity‐associated genes

The longevity‐associated genes (LAGs) are defined as genes whose genetic manipulation in model organisms (*M. musculus*, *D.  melanogaster*,* C. elegans* and *S. cerevisiae*) was shown to significantly affect their lifespan. The list was obtained from Human Ageing Genomic Resources (HAGR)–GenAge database (http://genomics.senescence.info/genes/longevity.html; Tacutu *et al*., 2013).

#### Interactome genes

Interactome genes were extracted from the BioGrid database (http://www.thebiogrid.org; Chatr‐Aryamontri *et al*., 2015) and were used as additional, high quality control for a more rigorous testing of the evolutionary conservation of LAGs.

#### Essential genes

Genes essential for the development and growth of *C. elegans* were extracted from WormBase (http://www.wormbase.org/; Howe *et al*., 2016).

#### Stress response genes

Genes classified under the category of ‘stress response’ were extracted by the UniProt Retrieve ID/Mapping service (http://www.uniprot.org/uploadlists/; Pundir *et al*., 2017)

### Determination of orthology

Ortholog determination for each gene was based on the InParanoid database—Eukaryotic Ortholog Groups (http://inparanoid.sbc.su.se/; Sonnhammer & Ostlund, 2015). The analysis was performed for 205 species (all species available excluding parasites; for a full list, see Table S1). The ortholog extraction was performed automatically using software developed in our laboratory. The taxonomy of the species examined was based on the ITIS database (http://www.itis.gov/). The statistical significance of conservation for a group of genes was evaluated with the chi‐squared goodness of fit test.

### Gene set enrichment

Enrichment analysis was performed using David Bioinformatics Resources 6.8 (https://david-d.ncifcrf.gov/; Huang *et al*., 2009), WebGestalt (http://www.webgestalt.org/; Wang *et al*., 2013), and EnrichR (http://amp.pharm.mssm.edu/Enrichr/; Kuleshov *et al*., 2016). The enrichment analysis was performed against three different backgrounds, including the whole genome, all LAGs, and the genes of the model organism under the same conservation criteria depicted in Tables [Link], [Link], [Link].

## Funding

This study was supported by the Fund in Memory of Dr. Amir Abramovich (to VEF), the Israel‐America Foundation (to VEF), the Israel Ministry of Science and Technology (to AB), and the EU funding through the Competitiveness Operational Programme 2014‐2020, POC‐A.1‐A.1.1.4‐E‐2015 (to RT).

## Conflict of interest

None declared.

## Author contributions

All authors participated in data collection and analysis. In addition, TB wrote all the programs for data extraction from the databases used and, in particular, the ortholog extraction for large sets of genes and species and the calculation of the conservation index. HY wrote the manuscript and prepared the figures and tables. AB participated in writing the manuscript. RT was involved in the computational aspects of analysis. VEF coordinated the study.

## Supporting information


**Fig. S1** Percentage of Interactome LAG orthologs from the four model species.
**Fig. S2** Percentage of LAG orthologs from the four model species after exclusion of proteins from enriched categories.
**Fig. S3** GO Slim summary and enrichment analysis.
**Fig. S4** Conservation index (CI) compared to concordancy of longevity effects.
**Fig. S5** Method similarity score compared to concordancy of longevity effects.

**Table S1** List of species used for orthology analysis.

**Table S2** Enrichment of KEGG and GOCC pathways in Longevity Associated Genes (LAGs) from different species.

**Table S3** List of proteins under different evolutionary conservation criteria.

**Table S4** Enrichment analysis of Longevity‐associated genes (LAGs) under different criteria and backgrounds.

**Table S5** Enrichment analysis of lifespan decreasing Longevity‐associated genes (LAGs) under different criteria and backgrounds.

**Table S6** Enrichment analysis of lifespan extending Longevity‐associated genes (LAGs) under different criteria and backgrounds.

**Table S7** List of LAGs that are listed as stress response genes under the GO classification system.

**Table S8** Pairwise concordance of LAG manipulation.
