# Supplementary material for: Wide‐scale comparative analysis of longevity genes and interventions
Source: Aging Cell. 2017 Aug 24;16(6):1267–75. doi: 10.1111/acel.12659 (PMC5676071; doi:10.1111/acel.12659)
Supplement: Supplementary file 1 — Fig. S1 Percentage of Interactome LAG orthologs from the four model species. Fig. S2 Percentage of LAG orthologs from the four model species after exclusion of proteins from enriched categories. Fig. S3 GO Slim summary and enrichment analysis. Fig. S4 Conservation index (CI) compared to concordancy of longevity effects. Fig. S5 Method similarity score compared to concordancy of longevity effects. [file ACEL-16-1267-s001.docx]

**Supplementary Figures and Tables**

**Suppl. Tables 1-8 are provided as additional Excel files.**


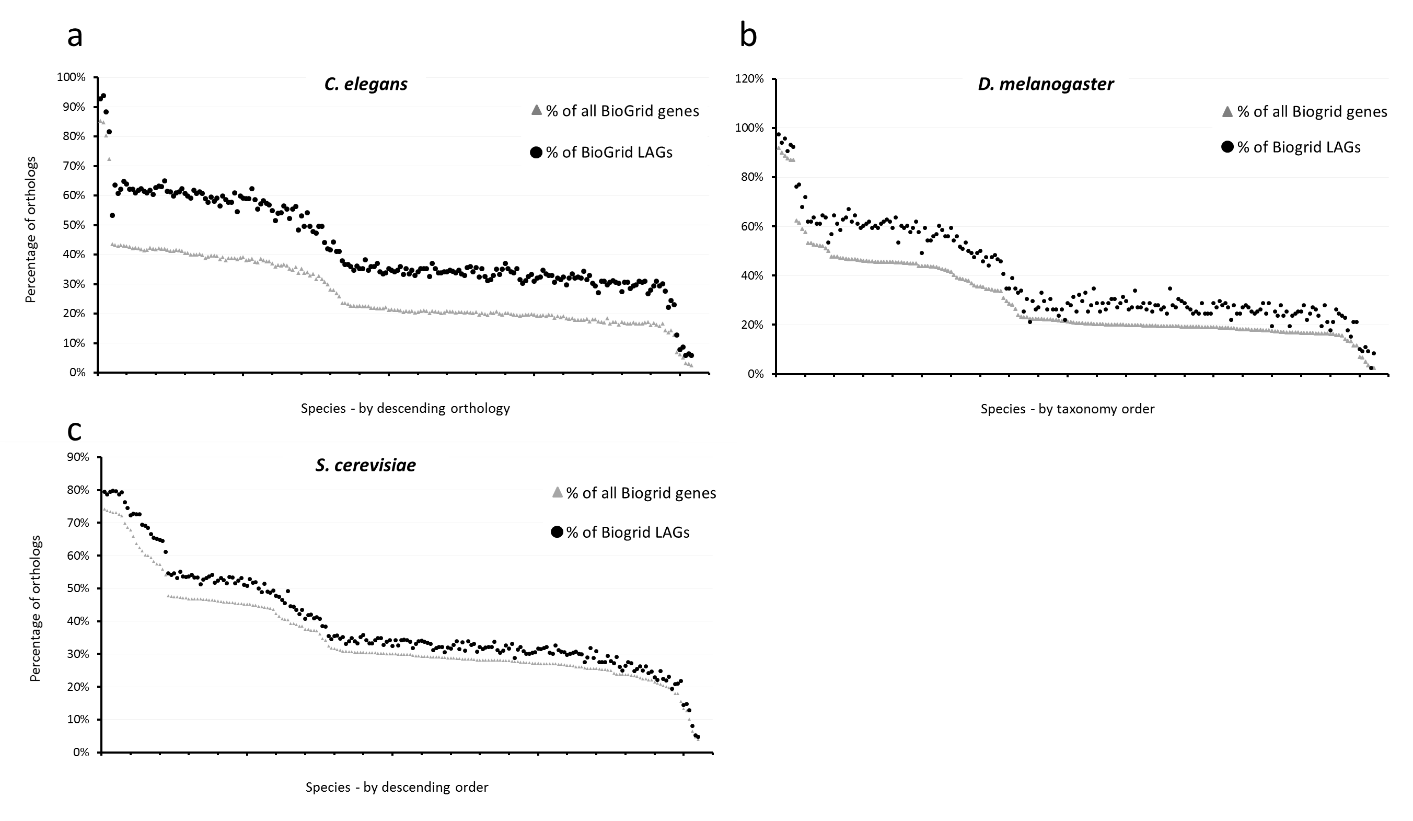


**Suppl. Fig. 1. Percentage of Interactome LAG orthologs from the four model species**. Each graph represents the LAGs discovered in the indicated model species. Each dot represents the percentage of orthologs between the model species and a different target species (total of 205 species from all kingdoms). Target species are ordered in descending order of orthology percentage as determined by the control. LAGs that are listed in BioGrid (black circle), entire proteome in BioGrid (grey triangle). **(a)** *C. elegans,* n = 3,865 for control and 343 for LAGs; **(b)** D. *melanogaster*, n = 8,026 for control and 118 for LAGs; **(c)** S. *cerevisiae,* n = 5,783 for control and 823 for LAGs.


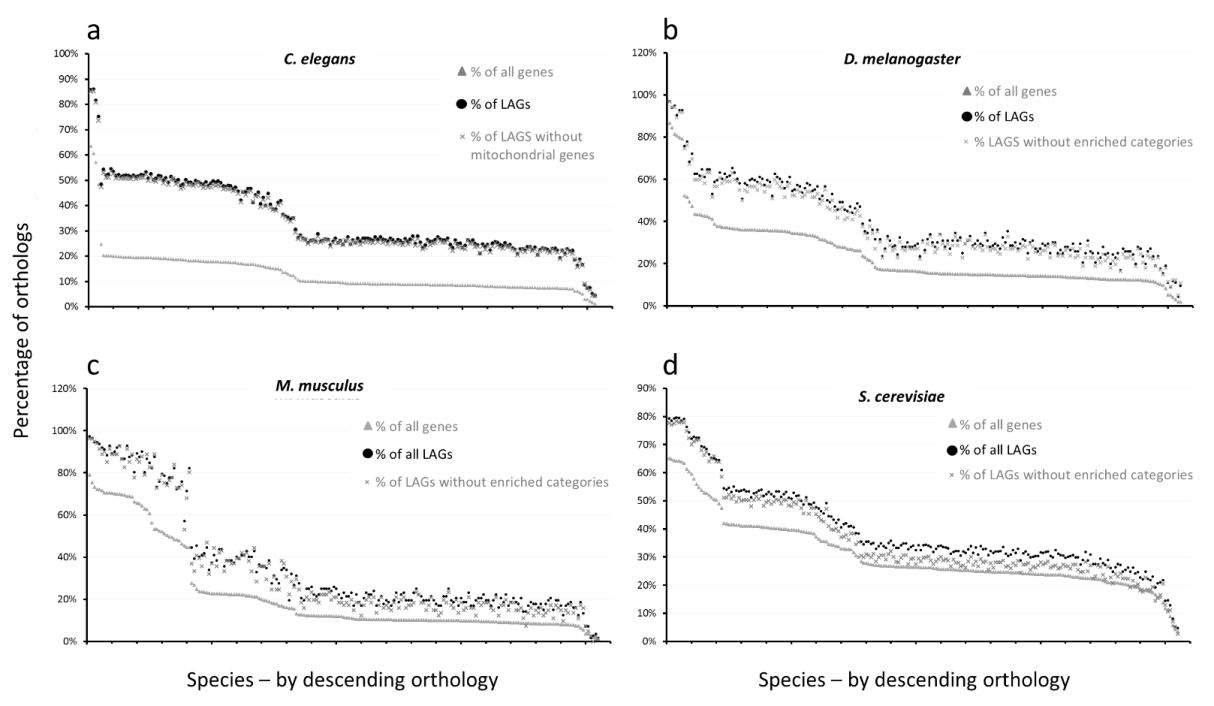


**Suppl. Fig. 2. Percentage of LAG orthologs from the four model species after exclusion of proteins from enriched categories**. Each graph represents the LAGs discovered in the indicated model species. Each dot represents the percentage of orthologs between the model species and a different target species (total of 205 species from all kingdoms). Target species are ordered in descending order of orthology percentage as determined by the control. LAGs (black circle), entire proteome (grey triangle) and LAGs after exclusion of proteins in enriched categories (grey x). (**a)** *C. elegans,* n = 20,325 for control, 733 for LAGs and 689 LAGs without mitochondrial genes; (**b)** *D. melanogaster*, n = 13,250 for control, 136 for LAGs and 122 for LAGs excluding enriched categories; (**c)** *M.* *musculus,* n = 21,895 for control,112 for LAGs and 73 LAGs excluding enriched categories ; (**d)** *S. cerevisiae,* n = 6,590 for control, 824 for LAGs and 551 LAGs excluding enriched categories. All differences presented are highly significant (p < 10^-10^).

a


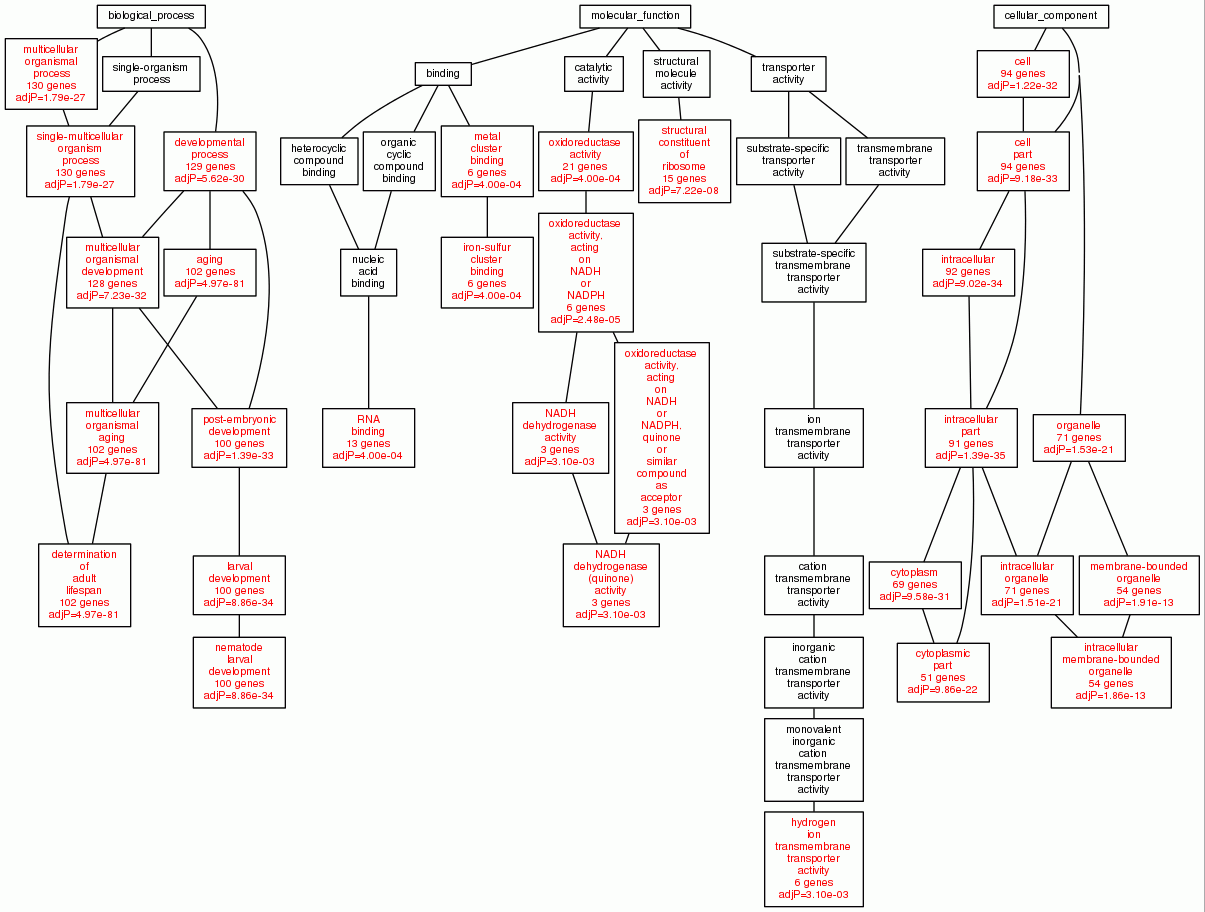


b


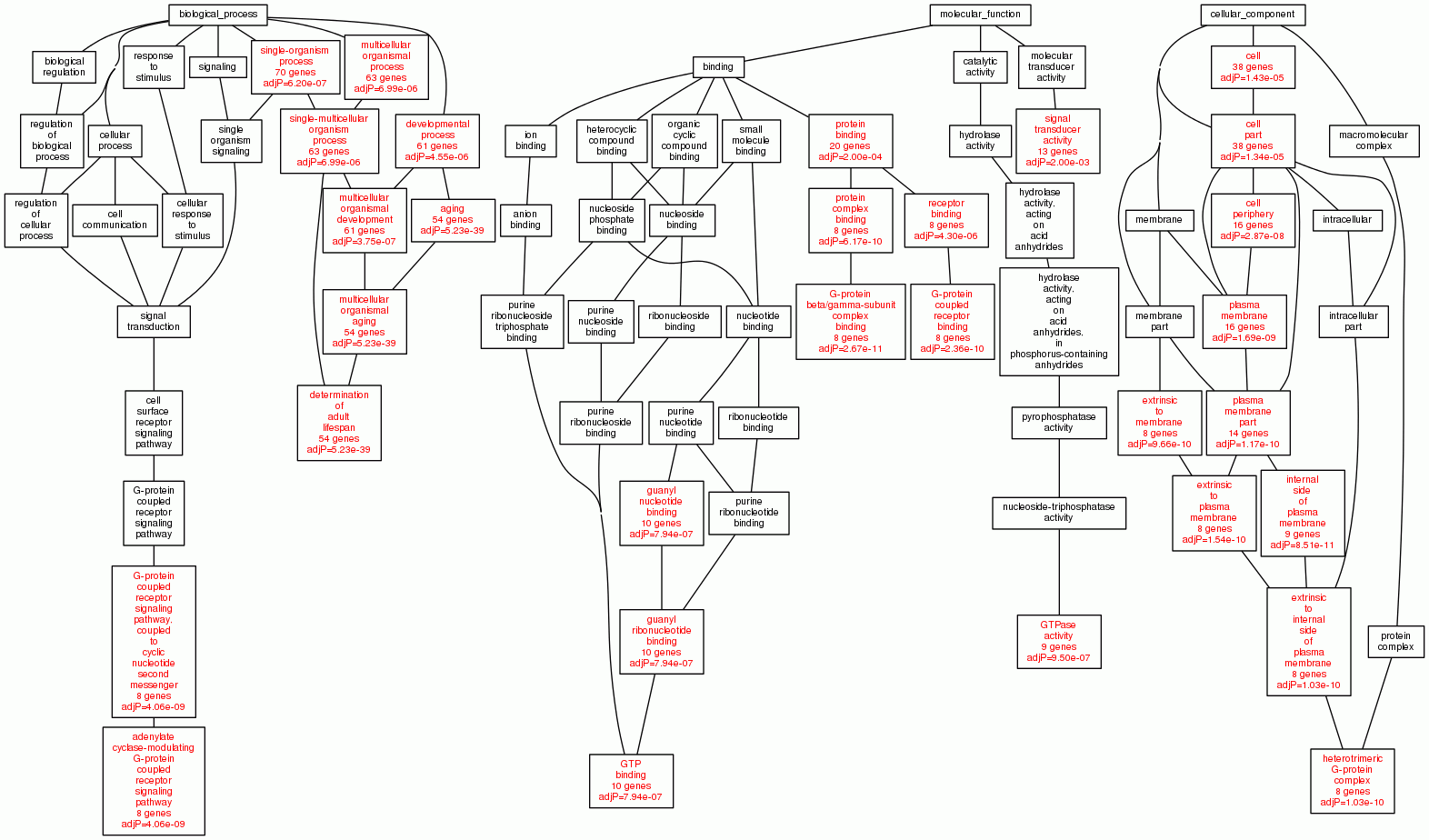


**Suppl. Fig. 3. GO Slim summary and enrichment analysis.** Presented are the results for the *C. elegans* “public” and “private” gene list enrichment analysis. The analysis was performed for the same gene lists as indicated in Suppl. Table 3, using the WebGestalt tool ((<http://www.webgestalt.org/>; Wang et al. 2013). **(a)** Public enriched categories (for at least 12 phyla). **(b)** Private enriched categories (only in Nematode).

**Suppl. Fig. 4. Conservation index (CI) compared to concordancy of longevity effects**. Each dot represents a pairwise comparison between orthologs of LAGs that were tested for their effect on longevity in more than one model species. The conservation index is the pairwise alignment score normalized to the protein amino acid length.

**Suppl. Fig. 5. Method similarity score compared to concordancy of longevity effects**. Each dot represents a pairwise comparison between orthologs of LAGs that were tested for their effect on longevity in more than one model species. The method similarity score was determined as: 0 = interventions of opposite directions (e.g. knockout and overexpression); 1 = intervention of the same direction but with varied methods (e.g. knockout and RNAi); 2 = interventions that are identical or very close to identical (e.g. knockout and knockout).
